# Efficacy and safety of tocilizumab (TCZ) in patients with systemic juvenile idiopathic arthritis (sJIA): TENDER 52-week data

**DOI:** 10.1186/1546-0096-10-S1-A58

**Published:** 2012-07-13

**Authors:** Fabrizio De Benedetti, Hermine Brunner, Nicola Ruperto, R Cuttica, Clara Malattia, Rayfel Schneider, Patricia Woo, Despina Eleftheriou, Eileen Baildam, Ruben Burgos-Vargas, Pavla Dolezalova, Stella M Garay, Rik Joos, Nico Wulffraat, Zbyszek Zuber, Francesco Zulian, Carine Wouters, Ricardo M Xavier, Lawrence Zemel, Stephen Wright, Andy Kenwright, Alberto Martini, Daniel Lovell

**Affiliations:** 1 Alder Hey Children's Foundation NHS Trust, Liverpool, UK; 2 Charles University in Prague, Prague, Czech Republic; 3 Children St. Louis Hospital, Strzelecka, Poland; 4 Cincinnati Children's Hospital Medical Center, Cincinnati, OH, USA; 5 Cincinnati Children's Medical Center, Cincinnati, OH, USA; 6 Connecticut Children's Medical Center, Hartford, USA; 7 Great Ormond Street Children Hospital, London, UK; 8 Hospital de Clínicas de Porto Alegre, Porto Alegre, Brazil; 9 Hospital for Sick Children, Toronto, ON, Canada; 10 Hospital General de Niños Pedro de Elizalde, Buenos Aires, Argentina; 11 Hospital General Mexico Univiersidad Nacional Autonoma Mexico, Mexico, Mexico; 12 Hospital IAEP, La Plata, Buenos Aires, Argentina; 13 Institute of Child Health, London, UK; 14 IRCCS G. Gaslini, Genova, Italy; 15 IRCCS Ospedale Pediatrico Bambino Gesù, Rome, Italy; 16 Roche, Welwyn, Hertfordshire, UK; 17 University Hospital Ghent, Ghent, Belgium; 18 University Hospital Leuven, Leuven, Belgium; 19 University Medical Center Utrecht, Utrecht, Netherlands; 20 University of Padua, Padua, Italy

## Purpose

Treatment options for sJIA are limited. Excessive IL-6 production has been implicated in several manifestations of this disease. In a previous Japanese study, TCZ, an IL-6 receptor inhibitor, improved arthritis and systemic features of patients with refractory sJIA. We present efficacy and safety of TCZ in patients with active sJIA who were treated for ≥52 wks in the global, 3-part, 5-yr, phase 3, multicenter TENDER study.

## Methods

Patients (N=112) 2–17 yrs with active sJIA for ≥6 mo and inadequate response to corticosteroids (CS) and NSAIDs were randomized 2:1 to TCZ (8 mg/kg if body weight ≥30 kg; 12 mg/kg if <30 kg) or placebo (control) every 2 wks for 12 wks in part 1; all patients received open-label TCZ at 8 or 12 mg/kg per body weight in part 2. Patients who escaped to open-label TCZ in part 1 also entered part 2. Oral CS tapering was permitted at wks 6 and 8 in part 1 and in the open-label extension in patients with ACR70 response, ESR <20 mm/h, and no fever. Efficacy data are presented for patients who had reached wk 52 of TCZ treatment by May 10, 2010 (n=88); safety data through May 10, 2010 are presented for all patients (n=112). Wk 52 baseline was the first TCZ dose; part 1 placebo patients were re-baselined when they escaped or entered part 2.

## Results

Proportions of TCZ patients who achieved JIA ACR30 + absence of fever or JIA ACR70/90 progressively improved from wk 12 to wk 52 (Table). Number (mean±SD) of joints with active arthritis or with limited range of motion decreased from 19.8±15.7 and 19.8±15.6, respectively, at baseline to 3.0±7.0 and 7.5±11.7, respectively, at wk 52, with 45% of patients having 0 active joints. At baseline, 55% of patients (n=62) had fever (temperature ≥37.5°C in the preceding 14 days), while at wk 52 only 9% (n=8) had fever. From baseline to wk 52, improvement in the scores was: CHAQ-DI from 1.7±0.9 to 0.7±0.8; physician global assessment VAS from 64.9±22.3 to 9.7±12.8, and patient/parent global assessment VAS from 58.7±24.4 to 12.6±18.5. There was a marked reduction in CS dose from 0.30±0.20 mg/kg/d at baseline to 0.06±0.08 at wk 52, with 48% having discontinued CS. There were 33 serious AEs (SAEs) in 25 patients; 12 SAEs were considered related (remote, possible, or probable) to TCZ (SAE rate: 0.23/patient year [PY] in part 1, 0.25/PY in part 2). Among the 15 serious infections, 6 were considered related to TCZ; all resolved and none led to study discontinuation. Twelve patients withdrew: 4 because of AEs; 4 because of insufficient response, 3 withdrew consent or did not return, and 1 died of a suspected tension pneumothorax unrelated to treatment.

**Table 1:** 

	Wk 12		Wk 52
	Control	TCZ	TCZ
JIA ACR responses, n (%)	(N=37)	(N=75)	(N=88)
ACR30 = absence of fever	9 (24)	64 (85)	77 (88)
ACR70	3 (8)	53 (71)	78 (89)
ACR90	2 (5)	28 (37)	57 (65)

## Conclusion

Year 1 results from this first global phase 3 study demonstrate that TCZ is highly effective and generally well tolerated in patients with sJIA.

## Disclosure

Fabrizio De Benedetti: Bristol-Myers Squibb, 5, Hoffmann-La Roche, Inc., 2, 5, Pfizer Inc, 5; Hermine Brunner: Roche , 5; Nicola Ruperto: None; R. Cuttica: None; Clara Malattia: None; Rayfel Schneider: Roche , 5; Patricia Woo: None; Despina Eleftheriou: None; Eileen Baildam: None; Ruben Burgos-Vargas: Abbott Laboratories, 5, 8, Pfizer Inc, 5, 8, Roche , 5, 8, Schering-Plough, 5, 8, Wyeth Pharmaceuticals, 5, 8; Pavla Dolezalova: None; Stella M. Garay: None; Rik Joos: None; Nico Wulffraat: None; Zbyszek Zuber: None; Francesco Zulian: None; Carine Wouters: None; Ricardo M. Xavier: Merck Pharmaceuticals, 8, Pfizer Inc, 5, 8, Roche , 8; Lawrence Zemel: None; Stephen Wright: Roche , 3; Andy Kenwright: Roche , 3; Alberto Martini: None; Daniel Lovell: Roche Diagnostics , 5.

